# Urban amenity and urban economic resilience: evidence from China

**DOI:** 10.3389/fpubh.2024.1392908

**Published:** 2024-05-09

**Authors:** Ran Du, Ke Liu, Dangru Zhao, Qiyun Fang

**Affiliations:** ^1^School of Economics, Huazhong University of Science and Technology, Wuhan, Hubei, China; ^2^International Business School, Shaanxi Normal University, Xi'an, Shaanxi, China

**Keywords:** urban amenity, economic resilience, livable city, population agglomeration, sustainable development

## Abstract

Under the influence of multiple uncertain factors at home and abroad, urban amenities, as the underlying support for urban renewal activities, are of great significance in enhancing urban economic resilience. The panel data of Chinese cities from 2011 to 2019 is used in this study. Urban amenity is measured from artificial amenities and climate amenities, respectively. By using a two-way fixed effects model, we empirically test the impact of urban amenities on urban economic resilience. The key findings of this study are as follows. (1) Urban amenities can significantly enhance urban economic resilience. (2) Heterogeneity analysis shows that there are regional differences in the role of urban amenities in promoting urban economic resilience, with cities in the eastern region, strong environmental regulations, and high urbanization rates benefiting more. (3) We further find that urban amenities mainly enhance economic resilience by promoting population agglomeration, attracting labor migration, improving the quality of human capital, and stimulating urban innovation. Our conclusions recommend to rationally allocate and optimize urban amenity resources, strengthen urban planning and construction management, and create a more livable urban environment, thereby enhancing urban economic resilience.

## 1 Introduction

Amid the complex backdrop of markedly increased internal and external environmental risks and the continued instability of the global economic situation, the Chinese economy is facing unprecedented challenges (1, 2). Enhancing economic resilience and ensuring stable economic operations have become critical issues that urgently need to be addressed in the process of achieving sustainable economic development (3). Urban economic resilience refers to the adaptability and resilience of the economic system of a city in the face of external shocks and changes (4), and is a concentrated expression of whether a country or region could effectively cope with the uncertainty shocks or resist and resolve economic risks. Improving the economic resilience of cities is conducive to the stability of the urban economic system and the high-quality development of the urban economy and society, which is an important focus of urban construction and development, and an inevitable choice for building a new urban development pattern. However, previous studies on the factors affecting urban economic resilience mainly focus on land, labor and capital (5–7). This model of improving urban economic resilience from the perspective of industrial production ignores the “human” factor and the essential needs of human beings, leading to problems such as urban environmental pollution, traffic congestion, too little public space, and shortage of leisure and entertainment facilities. These consequences could constrain the improvement of urban economic resilience. Particularly, China has experienced a rapid urbanization process, with hundreds of thousands of people moving from the countryside to the cities, which has led to a rapid expansion in the size of cities, and cities are facing great challenges in resource allocation and environmental pollution control (8). Researching urban amenities and economic resilience helps understand how Chinese cities perform in this dynamic change and contributes to the formulation of sustainable urban development strategies.

The Chinese government pointed out that the level of urban planning, construction and governance should be improved, and urban renewal actions should be carried out to build livable cities. In the context of China's new-type urbanization with “people” as the core and people's increasing expectations for a high-quality living environment, urban amenities and livability have become key elements to attract talents, promote innovation and cultivate emerging industries (9), and are directly related to the quality of life of urban residents and the efficiency of resource utilization in cities (10). Therefore, the construction of urban amenities not only concerns the quality of life and welfare of individuals but has also become a new driving force for enhancing the overall competitiveness of cities. It could have a positive impact on the long-term economic resilience of cities. Within the overall framework of urban growth theory, research on urban amenity can effectively address the deficiencies of traditional growth theories in explaining urban economic resilience, aiding city policymakers in the rational planning of urban comfort facilities. This approach could enhance local attractiveness and, consequently, improve long-term economic resilience. However, to date, there is scarce literature that delves into the impact of urban amenity on urban economic resilience from both theoretical and empirical perspectives. Therefore, in the context of increasing economic uncertainty, could enhance urban amenity lead to improved economic resilience in cities? What pathways and mechanisms facilitate this impact? Exploring these questions can expedite urban renewal activities, promote the construction of comfortable and livable urban environments, and provide policy direction and practical guidance for exploring ways to strengthen urban economic resilience.

The remaining part of this paper is organized as follows: Section 2 presents the literature review; Section 3 provides theoretical analysis and research hypotheses; Section 4 outlines the setting of the econometric model and variable explanations; Section 5 presents empirical analysis results; Section 6 concludes the paper and provides research insights.

## 2 Literature review and theoretical analysis

### 2.1 Literature review

With the development of information technology in the post-industrial era, cities have become not only carriers for economic benefits but also organic entities that meet the growing needs of residents. In this context, the theory of urban amenity has emerged. Foreign research on urban amenities mainly focuses on the connotation of urban amenities, the evaluation of urban amenities and its realistic value. Amenities are categorized into natural amenities, artificial amenities, and social atmosphere amenities based on the connotation of amenities. The study that urban amenities as pleasant living conditions that could promote population growth and economic growth (11). Some scholars believe that urban amenity also include various amenities that make residents feel physically and mentally pleased and attract them to live and settle in the city (12). Rogerson (13) argued that urban amenities not only enhance the happiness and satisfaction of city residents but also attract investors and innovative talents, serving as a novel external marketing strategy (13). In the evaluation of amenities, foreign studies mainly measure urban amenities through the following three methods. First, economists generally use housing prices and wage levels to reflect urban amenity, and use hedonic price models to quantify it (14). Second, a questionnaire is used to investigate the perception of comfort at the individual level (15). Third, conduct a comprehensive evaluation based on the connotation construction index of amenity items (16, 17). In addition, previous research has found that urban amenities can not only provide social value such as culture and art, but also promote urban economic development, thereby generating economic value (18). Currently, the research on urban amenities in China is mainly based on China's rapid urbanization process and the orientation of urban sustainable development policies, which provides an important research background. Research on urban amenities in China focuses mainly on two aspects: first of all, combined with the reality of China's development, the criteria for the construction of China's amenity system and the dynamic evolution process are proposed (19); secondly, empirically testing the economic effects of urban amenities on urban development, such as influencing land prices, increasing the degree of industrial agglomeration, and attracting labor inflow (20–22).

As the risks of uncertainty increasingly mount, the study of economic resilience has attracted widespread interest from scholars around the world. Davies explains economic resilience from three dimensions: first, the ability of an economy to withstand external risks and challenges, second, the ability of an economy to recover from negative shocks through self-regulation after the impact, third, the ability to innovate new growth pathways, enhancing the capacity for long-term growth (23, 24). Currently, there are various methods for measuring economic resilience, ranging from the sensitivity index method to comprehensive indicator evaluation methods (25–27). Urban economic resilience is thought to be determined by the dynamics of four interacting subsystems: the structural and business subsystem, the labor market subsystem, the financial subsystem, and the governance subsystem (4). Firstly, in terms of industrial structure and business system, existing scholars mainly study the impact of diversified or specialized industrial structure and technological innovation on economic resilience (5, 28, 29). Secondly, in labor market and financial subsystem, some scholars study the impact of human capital (30) and digital finance (23, 31) on economic resilience. Finally, there are also scholars discussing the influence factors of economic resilience from the perspective of governance such as policy support and political systems (32, 33).

Existing theoretical and empirical research on urban amenities and urban economic resilience provides a solid foundation for this paper. However, there are still two shortcomings. Firstly, while current studies have examined the impact of various factors on urban economic resilience, there has been less focus on the influence of urban amenities on urban economic resilience. Therefore, the relationship and impact mechanisms between urban amenities and urban economic resilience require further in-depth research. Secondly, the current methods for measuring different urban amenities levels need improvement. Existing studies often measure urban amenities from the perspective of guaranteeing indicators such as transportation, medical care, and the environment, with less consideration for social factors such as education and culture within the city. In the meanwhile, there is also less focus on the impact of climate amenities on urban economic resilience. Therefore, this paper takes 255 prefecture-level cities nationwide as the research objects, constructs artificial amenities and climate amenities, and studies the impact and internal mechanisms of urban amenities on urban economic resilience.

The marginal contributions of this paper may be reflected in three aspects. Firstly, existing research primarily focuses on the impact of industrial structure (6), technological innovation (5, 28), regional integration (7), infrastructures (34), digital finance (23, 31), and on economic resilience. Unlike previous studies that mainly focused on economic perspectives in studying economic resilience, this paper starts from the basic needs of “people,” using urban amenities as an entry point to explain how to enhance economic resilience. It supplements relevant studies on factors influencing urban economic resilience.

Secondly, previous studies have often selected indicators focusing on the natural environment and infrastructure to measure urban amenities (35, 36), with less emphasis on indicators related to healthcare and transportation. We chose a broader range of suitable indicators to assess the state of urban medical services and transportation infrastructure, thereby more comprehensively measuring urban amenities. Additionally, previous studies mainly used temperature and humidity indexes to study climate amenities (36, 37). In our study, based on the national standard for *Climatic Suitability Evaluating on Human Settlement* formulated by the Chinese government, we measure urban climate amenities more comprehensively, considering factors such as city temperature, humidity, wind speed, and sunlight duration. Thus, our construction of indicators more fully reflects both the artificial and climate amenities of cities, enhancing the accuracy of the indicators.

Thirdly, in terms of research content, we will identify external factors that differentiate the impact of urban amenities construction on urban economic resilience. This paper conducts a heterogeneity analysis from the perspectives of local environmental governance and regional development situations, expanding the directions in which urban amenities exert positive effects and the application scenarios of amenity theory, providing a new development approach for urban development models, offering more targeted support to comprehensively enhance urban economic resilience. Furthermore, from the perspective of urban population aggregation effects and innovation effects, we explore the potential impact mechanisms of amenities construction on urban economic resilience. Through comprehensive analysis of various channels, it helps identify the underlying logical impact.

### 2.2 Theoretical analysis and research hypotheses

Cities with more amenities can attract highly mobile resources such as capital, technology, and manpower, as well as foreign consumers and investors. This improves the city's competitiveness in acquiring these resources, thereby improving the urban quality and overall competitiveness as a comprehensive consumer product (38). This is beneficial for strengthening urban economic resilience. In addition, cities that are rich in amenities such as cultural institutions, educational facilities, and leisure spaces generally exhibit higher levels of economic diversification and complexity (39). The diversity of the economic system can help cities reduce their dependence on a single industry, enhance the city's ability to withstand stress, and enable cities to better cope with the risks of economic uncertainty. In an era of rapid urbanization, urban amenity has received attention for its key role in shaping urban development patterns and enhancing economic resilience.

#### 2.2.1 Urban amenity and population agglomeration effects

Firstly, according to the amenity migration theory, amenities in terms of natural environment, service environment, social culture, etc. are the main reasons for attracting population agglomeration and labor mobility. People tend to choose to move to cities with superior natural environments such as warm winters, cool summers, abundant sunshine, abundant green space and vegetation, and less pollution (40, 41). They also tend to move to cities with comfortable service environments such as diverse dining facilities, shopping malls, and efficient transportation infrastructure. At the same time, cities with more amenities develop faster and are more able to attract labor to move in, because cities with high amenity levels not only provide higher economic income but also provide a better quality of life (18). The process of population agglomeration in high-amenity cities will enhance urban economic resilience. On the one hand, the scale effect caused by population agglomeration can reduce transaction costs and create demand, support stable urban economic growth, and thereby enhance economic resilience. Population agglomeration can bring abundant labor resources to cities, provide enterprises with a variety of labor forces with professional skills, and reduce enterprises' labor search costs (29). It can also improve the allocation level of factor resources and improve economic efficiency, thereby enhancing urban economic resilience (30). Population agglomeration can also provide enterprises with a higher degree of talent adaptability, which can effectively avoid sharp changes in enterprise labor demand after economic shocks, quickly realize adaptive production structure adjustments, and thereby achieve sustainable economic resilience. In addition, as the population agglomerates in cities, it will expand the city's internal market demand, prompting industrial entities to produce more products. It will also prompt the government to increase infrastructure construction and improve the level of public services such as education and medical care, thereby reducing the impact of sudden changes in the external environment (17, 30). On the other hand, population agglomeration can also produce external effects. Population agglomeration will lead to the expansion and sharing of the labor market, which is conducive to increasing the degree of industrial agglomeration, promoting the refinement of the city's industrial division of labor, and forming an industrial structure system with complementary functions (42). The diversified industrial agglomeration has the function of an automatic stabilizer, which can effectively enhance urban economic toughness (43). Thus, urban amenities can increase population aggregation, thereby enhancing urban economic resilience.

Secondly, cities that offer a high quality of life, rich cultural experiences, a good social environment and diverse opportunities tend to attract more highly skilled people (44). Florida refers to the human capital that generates new technologies, knowledge and art as the “creative class,” which plays a decisive role in the innovative development of cities (45). Compared with low-skilled labor, high-skill “creative classes” are more sensitive to the living environment and working environment. A more comfortable environment can improve the work satisfaction and comfort of high-skilled talents, improve the efficiency of labor work, and reduce the loss of human capital (46). Multi-level and multi-skill labor can provide high-quality human capital for the professional industrial chain of the enterprise, and human capital is the key element of regional economic resilience construction (47). High-quality human capital can accelerate the flow and diffusion of innovative elements, release consumer domestic demand, and stimulate income effects, etc., promote the sustainable development of cities and enterprises (48). It gives cities the ability to adapt and resist external impact and enhance the urban long-term economic resilience (49). Therefore, cities with higher comfort standards such as culture and public services can attract the inflow of high-tech talents, and increase the local human capital stock, thereby promoting the high-quality development of cities and enhancing urban economic resilience (50).

#### 2.2.2 Urban amenity and innovation effects

As mentioned above, urban amenity is a key influencing factor of talent migration. Cities with higher levels of amenities are more able to attract innovative talents, and the comfort migration of talents can enhance urban economic resilience by stimulating innovation (51). Firstly, high-amenity cities can provide more space and facilities for relieving stress for innovative talents facing high work pressure, helping to create an inclusive and diverse social atmosphere, and providing greater social support for high-risk innovation (52). Secondly, a pleasant and comfortable environment, along with an innovative atmosphere, contributes to lowering the “talent entry barrier” (38). It encourages employees to showcase their abilities, enhances the enthusiasm for technological innovation among talents, attracts innovative companies and venture capital institutions, prompts cities to increase policy support for innovation resources, reduces innovation costs for relevant enterprises, and improves overall innovation efficiency. Finally, the concentration of innovative talents with diverse knowledge backgrounds in highly comfortable cities may lead to higher levels of technological spillover. Not only does this enhance the diversity and depth of technology, but it also encourages open and diverse innovative thinking, thereby expanding the boundaries of urban innovation (53). In addition, well-established infrastructures such as communication facilities can also facilitate technical exchange and information sharing among different cities, creating a diversified pool of technologies and a spillover effect of knowledge. The basis of promoting the efficient integration of traditional factors, drives the emergence of innovative schemes and products, providing businesses with more technological choices and higher-quality technological innovation (54). Furthermore, the technological innovation of cities also contributes to enhancing the city's economic resilience. On the one hand, innovation can improve the efficiency of resource allocation and the output efficiency of production factors. It can eliminate old production models and expand the scope of use of production factors, thereby deepening specialization, exerting the core driving force of market entities, and thus enhancing economic resilience (5, 55, 56). On the other hand, innovation can drive industrial structural transformation and upgrading, promote the transformation of industries toward rationalization, diversification, and sophistication, improve the overall division of labor and cooperation level of the urban economy, and enable cities to have a strong economic foundation to cope with adverse shocks (57). Therefore, urban amenities affect the spatial mobility of innovative talents, enhancing the level of urban innovation, and thus forming a city development model of “city amenity attracts talents, talents stimulate innovation, and innovation enhances resilience.”

Based on this, this paper proposes the following hypotheses:

Hypothesis 1: The improvement of urban amenities can enhance the city's economic resilience.

Hypothesis 2: Urban amenities can enhance economic resilience by promoting population agglomeration, attracting labor migration, and increasing the quality of human capital.

Hypothesis 3: Urban amenities can enhance the city's economic resilience by enhancing urban innovation.

**Figure 1:**
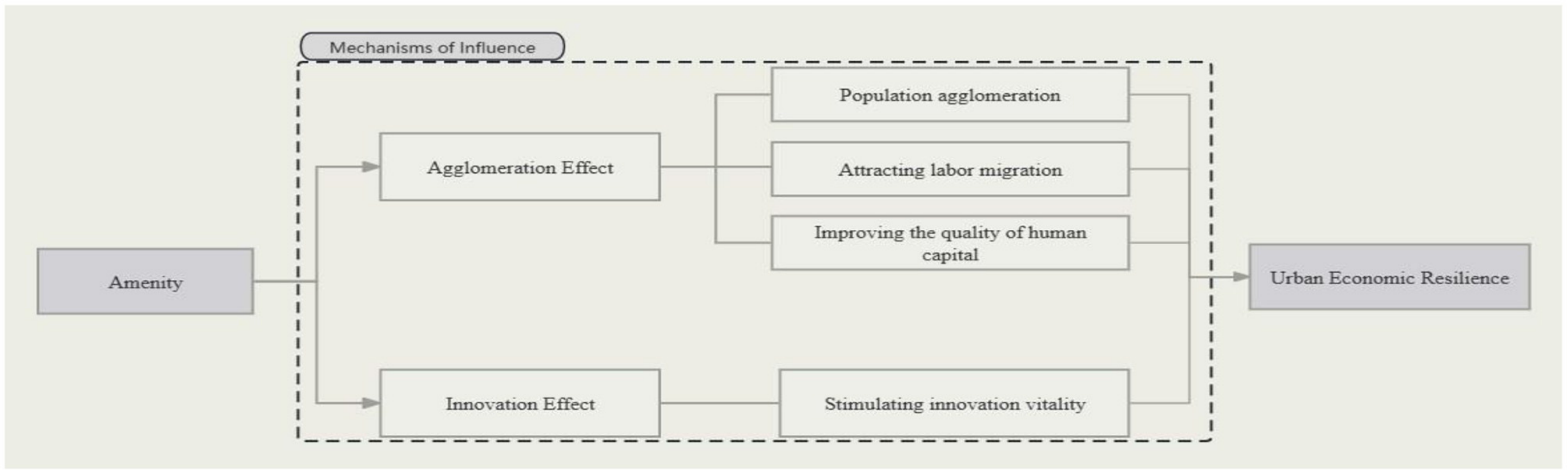
Influence diagram of the mechanisms by which urban amenity affects urban economic resilience.

In conclusion, the research framework is shown in Figure 1. It includes impact effects and impact mechanisms.

## 3 Methods and data

### 3.1 Empirical model

We control individual fixed effects and time-fixed effects. Individual fixed effects are used to control unobservable individual characteristic factors that do not change over time at the individual level but affect urban economic resilience. Time-fixed effects are time-characteristic factors that do not change with individuals but affect urban economic resilience. Fixed effects models can reduce endogeneity problems caused by omitted variables (58). We use Equation 1 to verify the impact of urban amenities on urban economic resilience.


(1) $$\begin{array}{l} Resilience_{it}=\beta_{0}+\beta_{1}Amenity_{it}+\underset{j}{\sum }\beta_{j}X_{ijt}+\mu_{i}+\delta_{t}+\varepsilon_{it} \end{array}$$

Where *i* and *t* represent the city and time, respectively, *j* represents the *j*-th control variable; *Resilience* represents the economic resilience of the city, *Amenity* represents the level of artificial amenities of the city, *X* represents control variables, μ_*i*_ represents city fixed effects, δ_*t*_ represents time fixed effects, and ε_*it*_ is the error term.

### 3.2 Variables

#### 3.2.1 Dependent variable

There are two main methods for measuring urban economic resilience. One is the indicator system method, which uses a series of indicators to measure economic resilience (59, 60). However, the indicator method has certain flaws. There is still no recognized reasonable indicator, and it is easy to confuse the causal relationship. The indicators used by researchers may be the reason why cities have economic resilience. Another method is to measure economic sensitivity indicators such as employment and GDP. Considering that the single indicator method has the characteristics of representativeness and continuity, we use this method to measure economic resilience (61). At the same time, considering that GDP is the core indicator of urban economic development, the sustained growth of urban GDP is the basis for solving a series of problems such as employment and welfare, and can directly reflect the ability of the urban economy to withstand shocks. Therefore, this article draws on Martin's economic sensitivity index and calculates urban economic resilience based on the ratio of the growth rate of urban regional GDP to the growth rate of China's national regional GDP (62). The city's economic resilience is evaluated by comparing the sensitivity index value with 1. If the sensitivity index is >1, then the city is more resilient and resistant to shocks than the national average, so the city is more resilient.

#### 3.2.2 Independent variable

At present, urban amenity is mainly measured by constructing an index system. Referring to the research of Diamond (46) and Zhang and Fang (20), our selects five major categories of indicators: culture, education, medical care, transportation, and environment, and uses the analytic hierarchy process to construct a comprehensive index that reflects urban artificial amenity (Amenity). We first constructed a hierarchical evaluation index system based on the principles of systematicness and availability of indicators. To avoid randomness in indicator selection, we fully draw on previous research and suggestions from experts in related fields. Secondly, use the upper-level indicators as the benchmark, compare each indicator at the same level, construct a judgment matrix, conduct consistency testing, and determine the weight of each indicator. Finally, according to the weighting method, the weight value of all indicators at this level to the previous level is calculated, and the comprehensive weight is obtained layer by layer. Previous literature also used the entropy weight method to calculate index weights, but the entropy weight method will lead to the loss of data information (63). Therefore, we use the analytic hierarchy process to determine specific weights. The five major categories of indicator data are all from the “China Urban Statistical Yearbook.” Table 1 reports the types and specific descriptions of the five major categories of indicators. We constructed the urban amenity variable based on these indicator systems.

**Table 1:** Selection of indicators for principal component analysis of urban artificial amenities.

**Amenity**	**Indicator**	**Description**	**Nature**
Artificial amenity	Culture	Number of Public Library Books per 10,000 People	+
		Percentage of Employees in the Cultural, Sports, and Entertainment Industry	+
	Education	Number of full-time teachers in elementary school	+
		Number of full-time teachers in general secondary schools	+
		Number of full-time teachers in general higher education	+
		Per capita expenditure on education	+
	Healthcare	Number of hospital beds per capita	+
		Number of doctors per capita	+
	Transportation	Urban road area per capita	+
		Number of public buses and trams in operation per capita	+
	Environment	Green space per capita	+
		Centralized treatment rate of sewage treatment plants	+
		Harmless treatment rate of domestic garbage	+
		Total environmental protection investment per capita	+

In the robustness test, we constructed a climate amenity index (Climate) based on the climate amenity evaluation standard for human settlements proposed by the China Meteorological Administration (GB/T 27963-2011) and replaced artificial amenity with climatic amenity as a proxy variable for urban amenity. The steps to construct climate amenities are as follows. The temperature and humidity index *I* and wind efficiency index *K* are calculated according to the following formulas (2) and (3), respectively. Based on the availability of data, the wind efficiency index is used in areas where the average wind speed during the evaluation period is >3m/s, otherwise, the temperature and humidity index is used. Finally, to ensure a consistent trend, according to the classification table of human settlement environment amenity levels in Table 2. Reassign “levels 1 and 5, levels 2 and 4, and level 3” to 1, 2, and 3, that is, the higher the value, the more comfortable the urban climate will be.


(2) $$\begin{array}{l} I=T-0.55\;*\left(1-RH\right)\;*\left(T-14.4\right) \end{array}$$

**Table 2:** Habitat amenities classification table.

**Level**	**Degree of feeling**	**THI**	**WCI**	**Description of feelings in healthy people**
1	Cold	< 14.0	< −400	Feeling very cold and uncomfortable
2	Cool	14.0−16.9	−400−300	Colder and less comfortable
3	Comfortable	17.0−25.4	−299−100	Feel comfortable
4	Hot	25.5−27.5	−99−10	Feeling hot, relatively uncomfortable
5	Sweltering	>27.5	>−10	Hot, uncomfortable, uncomfortable

We use Equation 2 to calculate the temperature and humidity index. Among them, *I* represents the temperature and humidity index, *T* represents the average temperature during the evaluation period, and *RH* represents the average relative air humidity during the evaluation period.


(3) $$\begin{array}{l} K=-\left(10\sqrt{V}+10.45-V\right)\;*\left(33-T\right)+8.55S \end{array}$$

We use Equation 3 to calculate the wind efficiency index. Among them, *K* represents the wind efficiency index, *T* represents the average temperature during the evaluation period, *V* represents the average wind speed during the evaluation period, and *S* represents average sunshine hours during the evaluation period. We construct an urban climate amenity index from this standard design.

#### 3.2.3 Control variables

When conducting empirical analysis, we refer to the research of Xu and Deng (56), Feng et al. (7), and Zhang et al. (64), and select economic scale, financial development level, fixed asset scale, industrial structure, fiscal expenditure and urban freight carrying capacity as control variables. 1. Economic scale (GDP). Cities with larger economies have stronger economic bases and resources in multiple dimensions, making them better able to adapt and respond to adverse economic conditions. This article uses the logarithm of GDP to measure the economic size of a city. 2. Financial development level (Finance). Cities with high financing potential are often able to attract more resources and investments, which can help cities build more resilient economies. We measure it by the ratio of deposit balances of financial institutions to GDP at the end of the year. 3. Scale of fixed assets (Investment). The scale of fixed assets is measured by the ratio of the city's total fixed assets to GDP. 4. Industrial structure (Structure). A diversified industrial structure can not only disperse the risks of economic shocks in a short period but also promote regional innovation and new technology innovation, allowing cities to adapt to resource reorganization and structural transformation and adjustment after the crisis. 5. Financial expenditure (Expenditure). Fiscal spending can improve economic resilience by stimulating demand, building infrastructure, and providing social protection. The city's financial expenditure is measured as the logarithm of the city's general fiscal budget expenditure. 6. Urban freight carrying capacity (Freight). The city's transportation infrastructure helps the city resist the impact of economic risks. The city's freight carrying capacity is measured by the logarithm of highway freight volume.

#### 3.2.4 Mechanism variables

We select the following variables as mechanism variables. 1. Population aggregation (Population). We measure the degree of population agglomeration using the logarithm of urban population density. 2. Labor supply and human capital. Labor supply (Labor) is measured by the ratio of the number of urban employees to the total population at the end of the year. Human capital stock (Humancap) is measured by the number of students in school. 3. Urban innovation. This article uses the following two indicators to evaluate urban innovation: the number of urban patent applications (Patent) and the level of R&D investment (R&D). The level of urban R&D investment is measured by the ratio of urban science education expenditure to GDP.

### 3.3 Sample

Considering the completeness of the data and the impact of public events, our study selects 255 cities in China from 2011 to 2019 as research samples. All macro-level data of cities are sourced from the “China Urban Statistical Yearbook.” The climate amenities indicators selected in this study include variables such as average temperature, average relative humidity, average wind speed, and average hours of sunshine for each city. The original meteorological data are obtained from the China Surface Climate Data Daily Value dataset (V3.0). We refer to the design method of Deschênes and Greenstone (65). We use the inverse distance weighted interpolation method (IDW) to interpolate daily meteorological data into grid data, and then obtain annual meteorological data for each district and county. To eliminate the influence of extreme values, all continuous variable data are trimmed by 1% above and below.

### 3.4 Probability density plot and descriptive statistics

**Figure 2:**
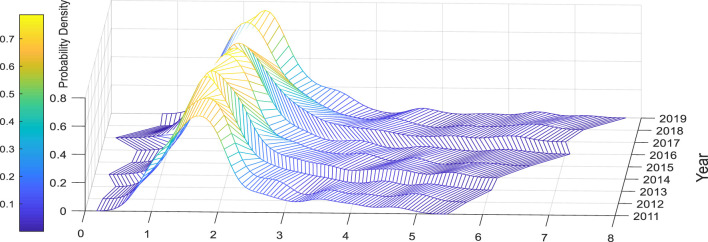
Probability density plot of amenity.

**Figure 3:**
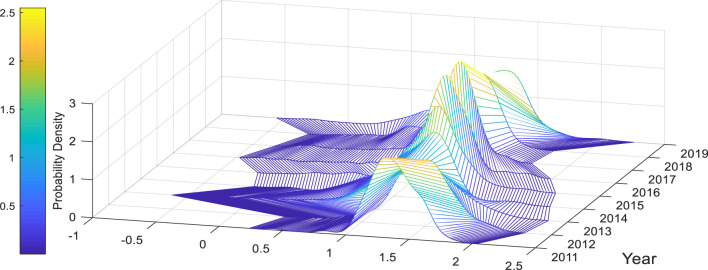
Probability density plot of resilience.

Figures 2, 3 respectively present the three-dimensional probability distribution diagrams of urban resilience and amenity of Chinese cities over the years. From Figure 2, it can be observed that the dispersion and right-skewness of the amenity probability distribution in Chinese cities are increasing year by year, the right tail is lengthening year by year, and the range is gradually expanding. The reason is that although the resilience values in the high quantile increase year by year, the values in the middle and low quantiles do not increase significantly, resulting in the gradual widening of the spatial gap in amenity among Chinese cities. From Figure 3, we can understand that the overall distribution of urban resilience in China fluctuates violently over the years, and the concentration ratio shows an “S”-shaped trend of first declining, then rising, and then falling again. Overall, there has been no significant improvement. Compared with other years, China's urban resilience was generally low in 2011, while in 2017 China's urban resilience was generally high. This may be due to the impact of the global financial crisis in 2008. China's resilience had not yet fully recovered in 2011. After 2015, China's implementation of supply-side structural reforms revitalized urban resilience. Secondly, we can also find that the spatial differences in urban resilience in 2019 are large, which may be due to the severe differences in urban resilience in China due to the Sino-US trade dispute in 2018. The above characteristics show that China's urban resilience still has room for optimization and improvement.

Descriptive statistics of variables are presented in Table 3. The mean value of resilience is 1.198, that is, the majority of urban economies demonstrate resilience. In addition, the average value of urban amenity is 2.295, the minimum value is 0.769, and the maximum value is 7.152, indicating that there is a significant difference in amenity levels in various cities.

**Table 3:** Descriptive statistics.

**Variable**	***N***	**Mean**	**Standard deviation**	**Min**	**Max**
Resilience	1,670	1.198	0.312	−0.307	2.036
Amenity	1,670	2.295	0.962	0.769	7.152
Climate	1,670	1.071	0.353	1	3
GDP	1,670	16.696	0.739	14.771	18.905
Finance	1,670	1.294	0.460	0.588	3.068
Investment	1,670	0.498	0.227	0.160	1.511
Structure	1,670	48.283	8.483	21.48	71.45
Expenditure	1,670	14.912	0.581	13.361	17.178
Freght	1,670	9.139	0.713	6.914	10.876
Population	1,670	5.863	0.831	2.351	8.207
Labor	1,670	0.117	0.077	0.035	0.640
Humancap	1,670	13.361	0.664	11.348	14.797
Patent	1,670	0.656	1.140	0.008	9.027
R&D	1,670	0.035	0.013	0.015	0.079

## 4 Empirical results analysis

### 4.1 Baseline regression results

The baseline regression results are shown in Table 4. Column (1) only includes core explanatory variables, and the results show that the improvement of urban amenities can significantly increase the economic resilience of a region. When control variables, city fixed effects, and year fixed effects are added in sequence from column (2) to column (5), the goodness of fit of the model is significantly improved. At the same time, the test results all show that the impact of urban amenities on urban economic resilience is significantly positive. As can be seen from column (5) in Table 4, after controlling for other factors, every 1-unit increase in urban amenities will increase urban economic resilience by 0.091 units. This result shows that the improvement of urban amenities can significantly enhance urban economic resilience. Economic resilience. Hypothesis 1 was verified.

**Table 4:** Baseline regression results.

**Variable**	**(1)**	**(2)**	**(3)**	**(4)**	**(5)**
	**Resilience**	**Resilience**	**Resilience**	**Resilience**	**Resilience**
Amenity	0.152^***^	0.032^**^	0.050^*^	0.037^***^	0.091^***^
	(5.448)	(2.254)	(1.757)	(2.832)	(3.472)
Control variables	No	Yes	Yes	Yes	Yes
Individual fixed effect	Yes	No	Yes	No	Yes
Time fixed effect	Yes	No	No	Yes	Yes
Observations	1,670	1,670	1,670	1,670	1,670
*R* ^2^	0.584	0.097	0.537	0.256	0.622

^***^, ^**^, and ^*^ indicate 1, 5, and 10% significance levels, respectively, with t-values in parentheses.

### 4.2 Robustness test

#### 4.2.1 Replacing the dependent variable

In the robustness test, we use two alternative indicators to measure the economic resilience of cities. The first method follows the research of Tan et al. (66), using China's annual actual GDP growth rate as the counterfactual basis for urban development, then calculating the difference between each city's annual actual GDP growth rate and this counterfactual basis, and using this difference to reflect the city's economic resilience level. This difference is used to construct a sensitivity index reflecting the level of economic resilience of cities. We put the sensitivity index into the benchmark model for regression, and the regression results are shown in column (1) of Table 5. The second method draws on the relevant research by Chen (67), using the regional employment sensitivity index to measure China's economic resilience and perform regression using the ratio between the change rate of urban employment and the change rate of national employment as the proxy explained variable. Regression is conducted with the ratio of the change rate of urban employment to the change rate of national employment as the proxy for the explained variable. The regression results are shown in column (2) of Table 5. The results all show that urban amenities can improve the economic resilience of the city, validating the baseline regression results.

**Table 5:** Robustness test.

**Variable**	**(1)**	**(2)**	**(3)**	**(4)**	**(5)**	**(6)**
Amenity	0.716^***^	3.007^*^		0.119^***^	0.050^**^	0.094^***^
	(3.550)	(1.695)		(3.979)	(2.372)	(2.798)
Climate			0.029^*^			
			(1.687)			
Individual fixed effect	Yes	Yes	Yes	Yes	Yes	Yes
Time fixed effect	Yes	Yes	Yes	Yes	Yes	Yes
Observations	1,670	1,337	1,670	1,520	1,621	1,169
*R* ^2^	0.637	0.163	0.619	0.618	0.603	0.684

^***^, ^**^, and ^*^ indicate 1, 5, and 10% significance levels, respectively, with t-values in parentheses.

#### 4.2.2 Replacing the independent variable

This study replaces urban artificial amenity with the climate amenity index. The empirical results are shown in column (3) of Table 5. The results show that climate amenities can significantly improve a city's economic resilience. The possible reason is that improved climate amenities are more conducive to attracting population agglomeration and business investment. Cities with a pleasant climate are usually rich in natural resources and ecosystem services, which not only provide urban residents with natural places for leisure and entertainment but also help improve their health and quality of life. High-quality ecosystem services can also attract more tourists and businesses, promoting the vigorous development of tourism and cultural industries. In addition, the improvement of investment attractiveness will help promote the city's infrastructure construction and industrial diversity development, thereby improving the city's overall economic level and enhancing the city's economic resilience.

#### 4.2.3 Excluding capital cities samples

As the administrative center of a province, provincial cities usually receive more government support and often have stronger resource attraction. These cities have large populations, high levels of economic development, complete infrastructure, and resource advantages in the construction of urban amenities. The interaction of these factors helps to improve the ability of provincial capital cities to cope with challenges and uncertainties, so the economic resilience of provincial capital cities may be stronger. Therefore, compared with non-provincial capital cities, provincial capital cities have a certain “siphon effect” in terms of resource acquisition and policy support, which may have an impact on the empirical results. To improve the credibility of the research conclusions, the empirical analysis was re-conducted after deleting provincial capital city data from all samples. Column (4) of Table 5 reports the results of regression on all non-provincial capital city samples after excluding provincial capital cities. A one-unit increase in urban amenities is associated with a 0.119-unit increase in economic resilience for non-capital cities. After provincial capital cities are eliminated, non-provincial capital cities without excess resources can also improve urban economic resilience through the construction of amenities. The results show that the impact of urban amenities in non-provincial capital cities on the city's economic resilience is still significantly positive, and the baseline regression is robust.

#### 4.2.4 Different samples and variable selection

We further select different samples and variables for robustness testing. Firstly, to further reduce the impact of extreme values on model estimation, the samples are winsorized at the 5% level, and the results are shown in column (5) of Table 5. Robustness results show that the core conclusions of this article still hold. Secondly, industrial agglomeration usually leads to the concentration of resources such as technology, talents, and raw materials, thereby forming a diversified industrial chain and economies of scale, which in turn helps the city respond to market changes and shocks and improve the city's economic resilience. In addition, FDI can introduce new technology and management experience and attract large amounts of capital investment, which is also important for the stable growth of the urban economy. Therefore, we add the proxy variables of industrial agglomeration and FDI to the control variables. This article selects the industry Herfindahl index to measure the degree of industrial agglomeration and uses the proportion of the total output value of foreign-invested enterprises in GDP to measure FDI. The results in column (6) of Table 5 show that after controlling for industrial agglomeration and FDI, the results are still significant.

#### 4.2.5 Endogeneity discussion

To reduce the problem of biased estimation results caused by reverse causality and omitted variables between urban amenities and economic resilience, this paper uses the instrumental variable method for empirical analysis. Drawing on the research of Xu and Deng (56), urban terrain slope was selected as the instrumental variable. First of all, terrain slope will have a significant impact on the construction form and investment costs of urban infrastructure such as roads, bridges, and parks. It also affects China's population distribution and labor concentration. The layout of infrastructure and population distribution are closely related to urban amenities. Therefore, there is a correlation between urban amenity and urban terrain slope, which meets the assumption of correlation between endogenous variables and instrumental variables. Secondly, the terrain slope is a naturally formed geographical information variable in the city. It has relatively natural exogenous characteristics and does not directly affect the current economic development level and resilience level of the city. It satisfies the homogeneity assumption of instrumental variables.

However, terrain slope is cross-sectional data in the data dimension and does not change with time, which will result in the inability to control the individual effects of cities in empirical regression. Secondly, the impact of the urban slope index on urban amenities may also change over time, and appropriate instrumental variables need to take into account this difference in time dimension. In addition, consider that urban wind speed may affect urban amenities and urban wind speed is determined by large-scale weather systems. Therefore, it is an exogenous factor in local economic activity. To this end, we multiplied the urban terrain slope (Slope) and the urban wind speed (Wind) and took the logarithm to construct an instrumental variable with a time effect.

Columns (1) and (2) of Table 6 are the instrumental variable regression results after adding only core explanatory variables, and columns (3) and (4) are the instrumental variable regression results after adding control variables. As can be seen from Table 6, the F values in the first stage are all >10, indicating that the instrumental variables and endogenous variables are related, eliminating the problem of weak instrumental variables. It can be seen from the regression results of the second stage that the coefficient of urban amenity is still significantly positive. This result is consistent with the baseline regression results and verifies the improvement effect of urban amenities on urban economic resilience.

**Table 6:** Instrumental variable analysis.

**Variable**	**(1)**	**(2)**	**(3)**	**(4)**
	**Amenity**	**Resilience**	**Amenity**	**Resilience**
Slope^*^Wind	0.083^***^		0.087^***^	
	(4.786)		(4.970)	
Amenity		0.416^*^		0.386^*^
		(1.859)		(1.816)
Control variables	No	No	Yes	Yes
Individual fixed effect	Yes	Yes	Yes	Yes
Time fixed effect	Yes	Yes	Yes	Yes
Observations	1,670	1,670	1,670	1,670
Stage I *F*-value		22.903		24.697

^***^, ^**^, and ^*^ indicate 1, 5, and 10% significance levels, respectively, with t-values in parentheses.

### 4.3 Heterogeneity tests

#### 4.3.1 Regional heterogeneity

Due to geographical differences, preferential policies, and other reasons, China's urban amenity construction and economic development levels have obvious regional differences. Therefore, there may also be differences in the role of urban amenities in promoting urban economic resilience. We divide the sample into eastern central and western cities for group regression. As shown in columns (1) and (2) of Table 7, for eastern cities, the improvement of urban amenities can significantly enhance urban economic resilience. The possible reason is that eastern cities usually have high population density, large market size, better infrastructure, and richer development resources. Secondly, eastern cities also have advantages in terms of openness and policy support, which results in the level of technology accumulation and the number of high-quality talents in eastern cities being far superior to that in central and western regions. Therefore, the more eastern cities can create a comfortable living environment through a series of policy measures, thereby enhancing urban economic resilience. The central and western regions are faced with problems such as weak economic foundations, lagging infrastructure construction, and serious population loss, which make them face relatively greater difficulties in improving economic resilience through urban amenity construction.

**Table 7:** Heterogeneity test.

**Variable**	**(1)**	**(2)**	**(3)**	**(4)**	**(5)**	**(6)**
	**East**	**Midwest**	**Strong environmental regulation**	**Weak environmental regulation**	**High-urbanized cities**	**Low-urbanized cities**
	**Resilience**	**Resilience**	**Resilience**	**Resilience**	**Resilience**	**Resilience**
Amenity	0.257^***^	−0.024	0.042^**^	0.026	0.130^***^	0.028
	(5.943)	(−0.785)	(2.505)	(0.466)	(3.197)	(0.691)
Control variables	Yes	Yes	Yes	Yes	Yes	Yes
Individual fixed effect	Yes	Yes	Yes	Yes	Yes	Yes
Time fixed effect	Yes	Yes	Yes	Yes	Yes	Yes
Observations	726	942	820	848	836	834
*R* ^2^	0.640	0.603	0.227	0.794	0.634	0.686

^***^, ^**^, and ^*^ indicate 1, 5, and 10% significance levels, respectively, with t-values in parentheses.

#### 4.3.2 Environmental regulation

Environmental regulation, as a means of government environmental governance, can effectively reduce environmental pollution problems, improve environmental quality, and enhance urban amenities. In addition, environmental regulatory policies can promote the growth of economic resilience by improving the level of urban technological innovation and promoting urban green transformation. Therefore, areas with strong environmental regulations may enhance the role of urban amenities in promoting urban economic resilience. Referring to the research design method of Chen and Chen (68), we selected the frequency of words related to environmental regulation in the “Government Work Report” of the prefecture-level city that year to measure the intensity of the city's environmental regulation. We grouped each city according to the median frequency of annual environmental regulation words and obtained the group with high environmental regulation intensity and the group with low environmental regulation intensity. Finally, the group regression analysis results are shown in columns (3) to (4) of Table 7.

#### 4.3.3 Degree of urbanization

There are significant differences in urbanization rates among different cities, which leads to diversity in the development patterns and speeds of various cities. We divide cities into cities with high urbanization rates and cities with low urbanization rates according to the median urbanization rate of each city. The results are shown in columns (5) and (6) of Table 7. The results show that when a city's urbanization rate is high, improvements in urban amenities will significantly enhance urban economic resilience. The possible reason is that the urbanization process is usually accompanied by the construction of infrastructure and the optimization of public services, including public transportation, public facilities, education, medical, and community services. High-quality infrastructure and public services can significantly improve the quality of life and satisfaction of urban residents, thereby enhancing the amenities of the city, promoting the development of the urban economy, and improving economic resilience. When the urbanization rate of a city is low, the concentration of production factors is usually relatively low, which may affect the improvement of economic benefits. At the same time, these cities have deficiencies in infrastructure construction and public service provision. Its ability to withstand various risks may also be weaker, affecting the city's economic resilience. Therefore, the urbanization process plays an important supporting role in improving urban amenity and economic resilience.

### 4.4 Mechanism analysis

According to the aforementioned theoretical analysis, urban amenities may have a significant impact on regional factor supply and resource allocation efficiency. High urban amenity means that the city has good living conditions, employment opportunities, educational resources, medical services, etc. These will increase people's expectations for the quality of life and happiness after migration, thereby increasing their utility expectations of migration. At the same time, areas with high urban amenities can attract high-quality talents in different fields or at different levels, further improving the quality of labor supply and improving the city's human capital level. Therefore, cities with high urban amenities can attract more population inflows and increase the quantity and quality of labor supply. In addition, cities with high urban amenities can also promote technological innovation and increase the supply of intellectual capital. Innovation is an important driving force for economic growth and a key factor in improving production efficiency and competitiveness. Therefore, high urban amenity means that the city has a good innovation atmosphere, scientific research institutions, talent training systems, knowledge exchange channels, etc. These will enhance the effects of population agglomeration and innovation, thereby enhancing urban economic resilience.

To test the channel through which urban amenity affects urban economic resilience, we take urban amenity as the core explanatory variable and replace the explained variables of the model (1) with the degree of population agglomeration, the number of labor forces, human capital, and urban innovation. This model is used to examine the impact of urban amenity on mechanism variables. Columns (1) to (5) of Table 8 respectively verify that the degree of population agglomeration, the number of labor force, human capital, innovation patents, and innovation investment are the paths through which urban amenity affects urban economic resilience. In conclusion, cities with high levels of amenities have a higher quality of life, more labor and high-quality talent inflows, and a stronger innovation atmosphere, which have a positive effect on urban economic resilience. The above analysis has verified to a certain extent the impact mechanism of urban amenities on urban economic resilience.

**Table 8:** Mechanism analysis.

**Variable**	**(1)**	**(2)**	**(3)**	**(4)**	**(5)**
	**Population**	**Labor**	**Humancap**	**Patent**	**R&D**
Amenity	0.038^***^	0.014^***^	0.051^***^	0.610^***^	0.001^***^
	(7.808)	(2.803)	(4.146)	(7.277)	(2.663)
Control variables	Yes	Yes	Yes	Yes	Yes
Individual fixed effect	Yes	Yes	Yes	Yes	Yes
Time fixed effect	Yes	Yes	Yes	Yes	Yes
Observations	1,670	1,670	1,670	1,670	1,670
*R* ^2^	0.999	0.929	0.987	0.900	0.952

^***^, ^**^, and ^*^ indicate 1, 5, and 10% significance levels, respectively, with t-values in parentheses.

## 5 Discussion

We analyze the relationship between urban amenities and urban economic resilience. Our empirical evidence suggests that urban amenities significantly contribute to enhancing urban economic resilience. This aligns with prior research emphasizing the various benefits of amenities in urban sustainable development (16, 21, 69). Urban amenities, encompassing factors such as green spaces, cultural attractions, and public infrastructure, not only enhance the quality of life for residents but also attract investments, stimulate economic activity, and promote sustainable development. The presence of green spaces, for instance, not only enhances environmental sustainability but also fosters community wellbeing and social cohesion, thereby contributing to enhancing urban economic resilience (70). Similarly, investments in cultural institutions and recreational facilities not only enrich the urban experience but also promote economic development through tourism, cultural events, and creative industries (71). In particular, we found that the population agglomeration effect and innovation effect are the influencing mechanisms through which urban amenity improves urban economic resilience. Population agglomeration and innovation effects play a very important role in enhancing the economic resilience of urban areas (64, 67). Concentration and innovation of population facilitate economies of scale, resource sharing, and enhanced labor market efficiency, all of which contribute to the resilience of local economies. In conclusion, investing in the development and maintenance of urban amenities emerges as a strategic approach to improving economic resilience.

The regional disparities in the impact of urban amenities on economic resilience are noteworthy. Previous studies find that unreasonable investments in amenities in poor regions could exacerbate socioeconomic inequalities, thereby leading to the exclusion of underprivileged residents from benefiting from urban amenities and displacing long-standing communities (72). This highlights the importance of tailoring urban development strategies to regional contexts. Similar to previous studies, we find that in more economically developed areas such as the East, the construction of infrastructure and other amenities is more conducive to promoting urban economic development (73, 74). Economically developed regions tend to have more diversified economies, meaning they are not solely reliant on one industry. Building amenities can contribute to this diversification by attracting different types of businesses and residents. For instance, a city with a vibrant arts scene may appeal to creative professionals, while excellent recreational opportunities may attract outdoor enthusiasts or retirees. This diversity can help protect the city against economic shocks. In addition, we also found that the stronger the urban environmental regulation, the more conducive it is to enhancing economic resilience through the construction of amenities. Environmental regulations targeting climate change mitigation and adaptation measures can enhance urban resilience to cope with extreme weather events and other economic-related challenges. Investments in green infrastructure, such as flood protection systems and sustainable urban planning, can minimize economic disruptions caused by environmental disasters (75). Therefore, policymakers should consider local conditions, socio-economic dynamics, and environmental factors when planning and allocating resources for urban amenities.

Previous research shows urban growth and economic development have not coincided with urban resilience policies, plans, and practices (76). Urbanization, especially in developing nations, is often characterized by rapid expansion and resource-intensive development aimed at bolstering economic growth. However, this growth frequently occurs without adequate consideration for the resilience of urban systems to withstand various shocks, ranging from environmental disasters to economic downturns. Consequently, cities may become more susceptible to disruptions, thereby impeding sustained economic progress. Our research underscores the significance of integrating resilience-focused approaches into urban development strategies, particularly through the adoption of suitable amenities. By investing in infrastructural solutions that prioritize both economic growth and economic resilience, cities can better withstand and recover from adverse events while fostering long-term prosperity. In conclusion, our study underscores the importance of aligning urban development efforts with economic resilience principles to promote sustainable economic growth in developing countries. By the construction of rational and moderate amenities, cities can enhance their capacity to withstand and recover from challenges, ultimately fostering more resilient and prosperous urban environments.

## 6 Conclusion

At this stage, economic uncertainty is becoming normalized. Increased economic uncertainty may increase market risks and trigger economic turmoil, which will have a profound impact on urban economic development. Therefore, how to deal with economic uncertainty and improve the economic resilience of cities has become an important current research topic. This is related to the efficiency of economic operation and the strategic goal of high-quality development of the Chinese economy. At the same time, urban amenity is an important indicator to measure people's happiness. The construction of urban amenities is an important task that conforms to the laws of urban development and the requirements of the times and reflects the comprehensive strength of the city. This is not only an effective way to deal with economic uncertainty, but also an important measure to achieve high-quality urban development. After measuring the city's artificial amenities and climate amenities, we conducted an empirical analysis based on China's urban panel data from 2011 to 2019, tested the impact of urban amenities on urban economic resilience, and came to the following empirical conclusions. Firstly, whether urban amenity is measured in terms of artificial amenities or climate comfort, cities with more amenity show stronger economic resilience when facing external shocks. Secondly, the impact of urban amenity on urban economic resilience shows regional differences. Compared with central and western cities, eastern cities can significantly enhance the city's economic resilience by improving their amenity. At the same time, the greater the intensity of environmental regulation and the higher the degree of urbanization in a city, the stronger the role of urban amenities in promoting economic resilience. Finally, we find that urban amenity affects economic resilience mainly through mechanisms such as population agglomeration, labor migration, improving the quality of human capital, and stimulating innovation vitality.

In light of the above conclusions, we propose the following policy recommendations: First, we suggest strengthening the incentives for local governments to build livable cities and, based on urban development goals and residents' needs, conduct reasonable allocation and optimization of amenity resources. The government should enhance residents' awareness of participation in urban governance, establish surveys on urban residents' satisfaction with livability, and incorporate urban livability satisfaction into the assessment and evaluation of government performance. Additionally, the government should delve deeply into and make good use of urban cultural resources, creating distinctive cultural blocks and cultural industry parks, among others. In the meanwhile, it should improve the allocation of urban land resources, dedicating more land to the construction of parks, green spaces, sports, and fitness facilities, and other public spaces. Lastly, according to the climate characteristics of different cities, the government should actively create green development space, optimize the urban heat island effect, strengthen environmental governance and ecological restoration, in particular, strictly implement the discharge standards of air pollutants and urban sewage and environmental protection policies, improves the quality of air and water resources, and improves the urban climate environment.

Second, considering the differences in natural conditions and the level of economic and social development between different regions, the government should promote the construction of urban amenities in a manner tailored to local conditions. In the eastern regions and cities with higher urbanization rates, it's crucial to fully leverage the advantages of factor agglomeration and location. The focus should be on improving urban environmental quality and public service levels. By intensifying environmental governance, advancing green and low-carbon development, optimizing urban spatial structure, and enhancing urban management efficiency, the goal should be to create ecologically livable, modern, resilient cities. In addition, the government should pay attention to the regional balance of the allocation of amenity resources, improve the financial transfer payment system, and increase the tilt of amenity resources in the central and western regions and cities with low urbanization rates. Governments at all levels should improve the resource scheduling mechanism for amenity construction and increase special support for these areas such as funds. In the meanwhile, the government should promote the central and western regions and cities with low urbanization rates to invest appropriately in infrastructure construction according to the needs of the population, strengthen the development of the value of natural resources, and foster tourism, leisure, and health care industries. The government should narrow the differences in education, medical, and other resources between regions, and strengthen exchanges between cities in school management, curriculum education, and student training. In particular, it should solve the problem of remote medical care and difficult access to medical care, guide the rational flow of the population, and thus enhance the economic resilience of regional cities.

Third, the government should improve the overall environmental quality of the city, attract population and capital inflows, and stimulate the quality of urban innovation. First and foremost, the government should prioritize human-centric improvements in urban public services and infrastructure, enhance urban green spaces, air quality, and other environmental aspects, foster a shared local culture, and judiciously promote unique cultural products in crafts and cuisine to fully accommodate the diverse needs of various demographics and activities. Concurrently, it should establish a tiered housing system to enhance living comfort, expedite the development of affordable housing, and bolster community governance capabilities, all aimed at forging a conducive environment for work, living, leisure, and travel, thereby attracting populations and labor migration. Moreover, the government should ease household registration constraints, diminish labor mobility barriers, and fortify the urban social welfare system, crafting an open, inclusive, and diverse social milieu. Lastly, the government ought to underscore the significance and timing of constructing various amenities, forge an inclusive environment for innovative talents, and high-caliber research platforms, actively recruit and nurture top-tier talent, bolster the growth of diverse innovative bodies, and facilitate the translation and deployment of scientific and technological advancements to fortify urban economic resilience.

The shortcomings and future development ideas of this paper are as follows: First of all, we mainly use objective index data at the city level. Future studies could further consider the use of subjective evaluation index data of urban residents on the amenity level or individual characteristics data at the micro level, and use Hedonic or other models to estimate the implied price of urban comfort attributes and improve the comfort index construction system. Secondly, the measure of economic resilience in this paper has limitations in the selection of variables and the evaluation of effects and lacks the dynamic evaluation of the time dimension. In the future, multiple macroeconomic indicators and time-varying impulse response functions could be selected and used to measure urban economic resilience, to provide ideas for measuring macroeconomic resilience from a dynamic perspective.

## Data availability statement

The original contributions presented in the study are included in the article/supplementary material, further inquiries can be directed to the corresponding author.

## Author contributions

RD: Conceptualization, Formal analysis, Funding acquisition, Software, Writing – original draft, Writing – review & editing. KL: Writing – original draft, Writing – review & editing. DZ: Formal analysis, Software, Writing – review & editing. QF: Conceptualization, Writing – review & editing.
